# Moderate heating of waterline improves dental unit water quality by activating bactericidal properties of tap water

**DOI:** 10.1016/j.jds.2025.05.023

**Published:** 2025-06-06

**Authors:** Kunihiro Fushimi, Masahiro Yamada, Jun Watanabe, Jumpei Washio, Nobuhiro Takahashi, Hiroshi Egusa

**Affiliations:** a Division of Molecular and Regenerative Prosthodontics, Tohoku University Graduate School of Dentistry, Sendai, Japan; b Division of Mechanobiology and Biomedical-Dental Engineering, Tohoku University Graduate School of Biomedical Engineering, Sendai, Japan; c Division of Oral Ecology and Biochemistry, Tohoku University Graduate School of Dentistry, Sendai, Japan; d Global Strategy Office, Tohoku University, Sendai, Japan; e Center of Excellence for Dental Stem Cell Biology, Faculty of Dentistry, Chulalongkorn University, Bangkok, Thailand

**Keywords:** Free residual chlorine concentration, Healthcare-associated infections, Heterotrophic bacteria, Water quality management

## Abstract

**Background/purpose:**

Daily flushing of dental unit waterlines (DUWLs) with fresh tap water for an adequate duration each morning before dental procedures is essential to prevent healthcare-associated infections. However, the bacterial reduction achieved by flushing alone is often temporary and may be insufficient. The purpose of this study was to evaluate the management practices of clinically used DUWLs and identify effective measures for improving water quality.

**Materials and methods:**

The bactericidal free residual chlorine concentration (FRCC) and heterotrophic plate counts (HPCs) in air turbine handpiece DUWLs with or without chemical disinfectants and/or heating apparatus were evaluated before and after flushing, based on legal standards and target values for water quality assessment.

**Results:**

Residual water in the DUWL consistently exhibited lower FRCC and higher HPCs than the legal standard and target values, respectively. Extended flushing in water discharged from conventional dental units increased FRCC beyond the legal standard value; however, HPCs did not consistently decrease below the legal target value. Flushing DUWLs equipped with a chemical disinfectant apparatus reduced HPCs to below the legal target value but did not always restore FRCC completely. Notably, heating of DUWLs at 65 °C improved both FRCC and HPCs through flushing, regardless of the type of dental unit.

**Conclusion:**

Flushing plays a crucial role in maintaining the water quality of dental units. Moderate heating of DUWL can ensure compliance with legal standards for water quality management by enhancing the effectiveness of flushing.

## Introduction

In recent years, addressing healthcare-associated infections in dental clinics has become increasingly important. During treatment, handpieces disperse aerosols containing oral microorganisms throughout the operatory environment.^1^ Therefore, effective water quality control measures for dental unit waterlines (DUWLs) are essential. Water stagnation in inactive units lowers the chlorine concentration and promotes microbial growth.^2^ Several studies have reported infections linked to contaminated dental unit water, including a fatal case of Legionnaires disease in Italy,^3^ and an osteomyelitis outbreak in children in the USA.^4^ These cases mainly affect vulnerable individuals, such as the elderly and immunocompromised individuals. In Japan's super-aged society, in which many dental patients have systemic illnesses, the management of contamination in dental units is becoming increasingly critical.

Flushing is the most basic and widely used method for improving water quality.^5^ Although it is effective at reducing bacterial counts, its effects are short-lived.^6^ Other methods include filtration, ultraviolet irradiation, and tubing material modifications.^6^^,^^7^ Chemical disinfection using agents such as sodium hypochlorite, chlorine dioxide, chlorhexidine, hydrogen peroxide (H_2_O_2_), peracetic acid, and citric acid is commonly employed.^8^ These solutions are typically introduced during downtime, such as after clinic hours or on weekends.^6^ However, dental units without infection control systems are difficult to treat using disinfectants, and the chemical interactions between these agents and tap water components are not fully understood.

Heterotrophic bacteria form colonies under low nutrient and temperature conditions and serve as key indicators of water quality. Prior research has shown that moderate heating (60 ± 3 °C) reduces heterotrophic plate counts (HPCs) in water heater systems.^9^ Tap water contains free residual chlorine, which inhibits microbial growth and prevents biofilm formation.^10^^,^^11^ However, the free residual chlorine concentration (FRCC) tends to decrease with increasing temperature owing to volatilization.^12^ In a closed system such as a DUWL, the behavior of volatilized chlorine may differ from that in open systems. Therefore, combining moderate heating with flushing may enhance microbial control.

In this study, we hypothesized that both an external DUWL disinfection system and moderate heating could effectively improve the water quality of DUWLs. We examined the state of bacterial contamination in DUWLs and evaluated the impact of external chemical disinfection systems and moderate heating on water quality.

## Materials and methods

### Experimental design and water collection

Two types of conventional dental units (Refino and Eomregalo, GC Corporation, Tokyo, Japan) without built-in infection control were used. One of the two Eomregalo units was equipped with an external disinfection system (Twin Turbo Cleaner, GC Corporation), was referred to as the chemically disinfected unit. The Refino and the other Eomregalo unit, both lacking disinfection systems, served as the conventional unit. The Refino and Eomregalo units had been in operation since 2009 and 2012, respectively.

Each morning, approximately 200 mL of handpiece water and 800 mL of mouth rinse water in the Refino units were flushed according to the unit manual. For the Eomregalo units, all the water pathways were connected to a flushing tank, and tap water was circulated at a high flow rate for 7 min each morning before use. Pre-flushing was intentionally omitted for bacteriological evaluation, water quality surveys, and flushing analysis. Water samples (25 mL) were collected from seven points (Fig. 2A and B) along DUWL: (1) the main valve, (2) after the main valve, (3) before the unit branch within the unit, (4) cup filler, (5) inside the assistant table supplying water to the assistant's multi-syringe, (6) inside the doctor's table supplying water to the handpiece, and (7) the air turbine outlet. The samples were placed in sterile tubes to analyze of FRCC and HPCs.

**Figure 1:**
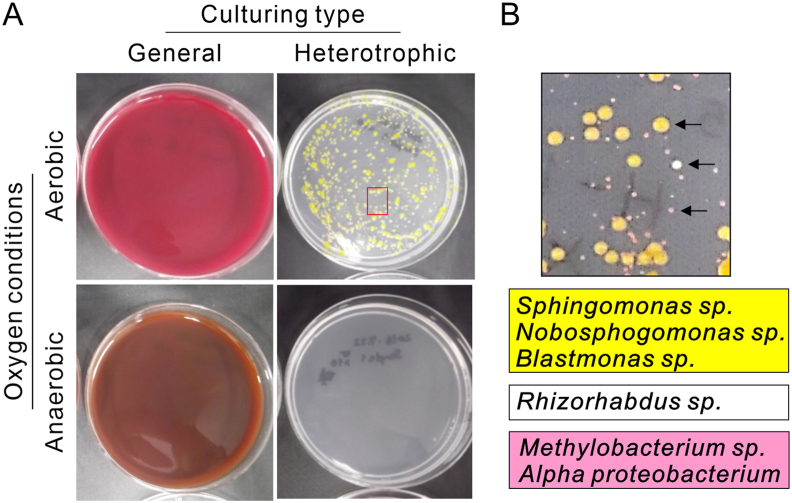
**Detection of general heterotrophic bacteria in the dental unit waterline.** (A) Images of blood and Reasoner's 2A (R2A) agar plate cultures showing general bacteria (left images) and heterotrophic bacteria (right images) in air turbine discharge water without flushing under aerobic (upper images) and anaerobic (lower images) conditions. No bacterial growth was observed on blood agar medium from the residual water of the dental unit waterline under either aerobic or anaerobic conditions, whereas a large number of heterotrophic bacteria were detected on R2A agar medium under aerobic conditions. (B) Higher magnification image highlighting the red rectangular region of the heterotrophic bacterial culture on the R2A agar plate in (A) and the corresponding list of bacterial species (sp.) detected in yellow, white, and pink colonies (indicated by black arrows). These species were identified by 16S rRNA sequence analysis. The background color of the list corresponds to the color of the detected colonies.

**Figure 2:**
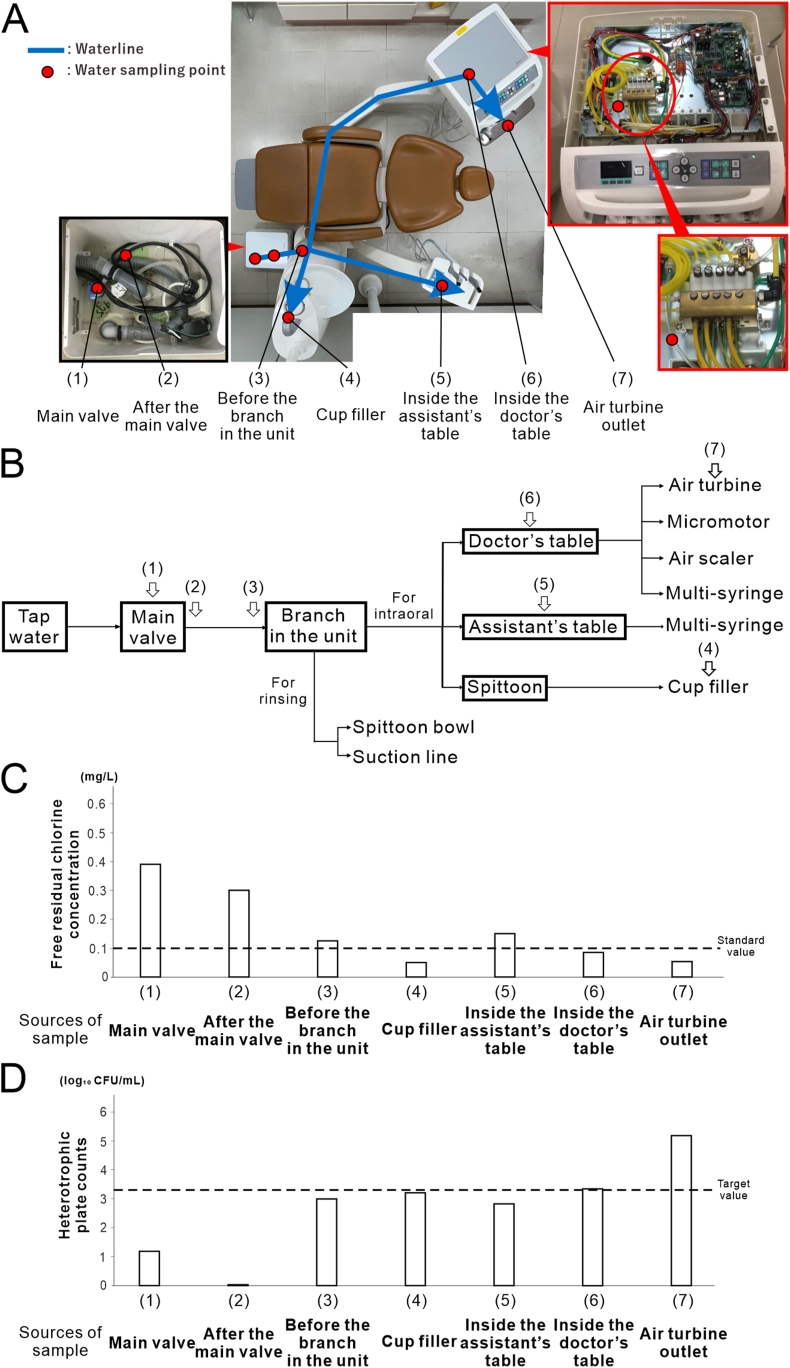
**Contamination of each part of the dental unit waterline.** (A) Conventional waterline dental unit. After entering the unit from the main valve, the waterline splits into two pathways: one supplying water for use in the oral cavity and the other for cleaning the equipment. The pathway for the oral cavity is further divided into three branches: the spittoon section supplying the cup filler, the assistant table, and the doctor table. Arrows indicate the direction of water flow, and the water sampling points are marked with red dots. The locations were numbered sequentially based on their distance from the main valve. (B) Schematic of the dental unit waterline, showing the water sampling points indicated by arrows. (C) Free residual chlorine concentration measured at each sampling point (*N* = 2). (D) Heterotrophic plate counts measured at each sampling point (*N* = 2)

25 mL samples were collected from the air turbine waterline before and after 1–4 min of flushing (each 25 mL corresponds to a 30-s flush) to assess the flushing efficacy. Additionally, 15 mL samples were collected from both units before and after 1–3 min of flushing to evaluate the chemical disinfection effects.

To examine the effect of moderate heating, 25 mL handpiece water samples were collected before heating. The heater integrated into the DUWL water tank (Fig. 5A) was set at 60–69 °C in 1 °C increments. After 30 s, 1 min, and 2 min of flushing, water temperatures were monitored at four points: the supply valve, inside the tank, tank outlet, and handpiece outlet using a four-channel digital logger (Memory HiLogger LR8431, Hioki E.E. Corporation, Nagano, Japan).

**Figure 3:**
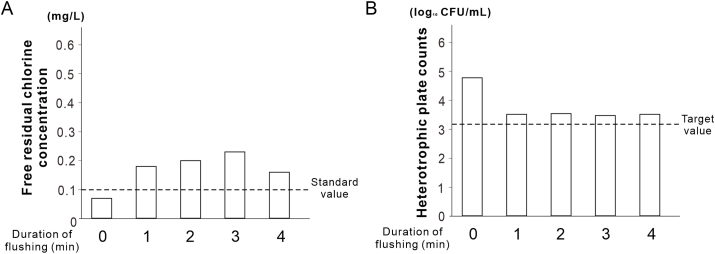
**Effect of flush duration on water quality in dental units.** (A) Recovery of free residual chlorine concentration in conventional dental units based on flush duration. (B) Reduction in heterotrophic plate counts in dental units as a function of flush duration. Data is shown as histograms (*N = 1*).

**Figure 4:**
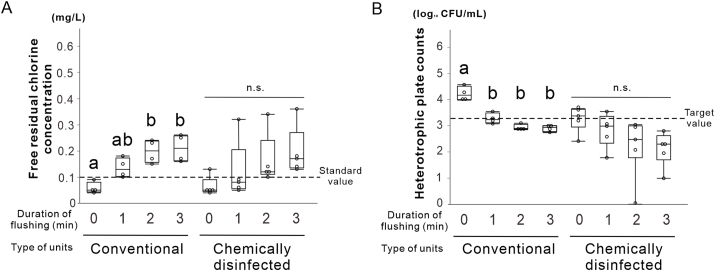
**Water quality assessment for the conventional unit and the unit with the external disinfecting equipment.** (A) Recovery of free residual chlorine concentration as a function of flush duration for each type of dental unit. (B) Reduction of heterotrophic plate counts as a function of flush duration for each type of dental unit. Data are shown as box plots (*N* = 4). Different letters in A and B indicate statistically significant differences between them (*P* < 0.05, Tukey's honest significant difference [HSD] test). n.s., non-significant difference.

**Figure 5:**
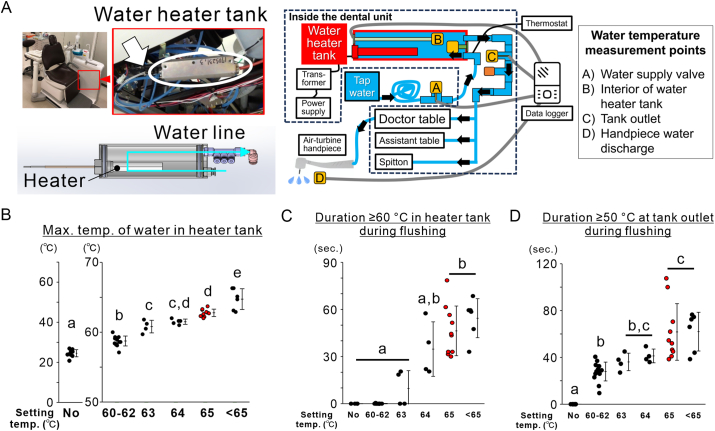
**Impact of moderate heating on the improvements to the handpiece water quality.** (A) Images of the heating device indicated by an arrow within a circle, and its location in the dental unit, and a computer-aided design image. Schematic showing the water temperature monitoring locations within the dental unit water line: water supply valve, interior of the water heater tank, tank outlet, and handpiece discharge point. (B) Maximum water temperatures recorded inside water heater tank at each temperature setting. (C) Duration above 60 °C in the heater tank during flushing. (D) Duration above 50 °C at tank outlet during flushing. Data are shown as dot plots with means and standard deviations (*N* = 3–10). Different letters indicate statistically significant differences (*P* < 0.05, Tukey's honest significant difference [HSD] test).

For heat validation, purified deionized water (MonotaRO Co., Hyogo, Japan) was introduced at 4 kgf/cm^2^ from a pressure tank (Mister AUTO HS-503W; Koshin Ltd., Kyoto, Japan). After the last clinical session, the DUWLs were filled and flushed three times in the conventional unit equipped with the heater. Water was sampled the following Monday morning. Control samples (15 mL) were collected from the purified water supply. The post-heating water temperature was monitored using a digital logger.

### Chemical disinfection

For the chemically disinfected unit, a 3 % H_2_O_2_ solution with silver ions (Twin Turbo Cleaner Solution, GC Corporation, Tokyo, Japan) was diluted 30-fold, circulated through the DUWL, and left to stand for two days each week. Routine flushing was performed every morning before use.

### Measurement of FRCC

The FRCC was measured using a N,N-Diethyl-p-phenylenediamine (DPD) sulfate salt colorimetric assay (DPD Plus, Oyalox Co., Tokyo, Japan) and a residual chlorine analyzer (Photometer CL, Oyalox Co.).^13^ After a 30 s reaction, measurements were conducted within 1 h of sampling. FRCC levels were compared against the Japanese water quality standard (≥0.1 mg/L).

### Bacterial plate count

To assess hemolytic bacteria, samples were cultured on blood agar (BD BBL™, 5 % sheep blood, Becton Dickinson Japan, Tokyo, Japan) at 37 °C under aerobic and anaerobic conditions for seven days.^14^^,^^15^ For HPCs, 100 μL of water was inoculated on R2A agar (BD BBL™) and incubated aerobically at 20 °C for seven days. Plates with 30–300 colonies were counted, and no growth was recorded at 1 CFU/mL. The results were evaluated against the Japanese control target (<3.30 log_10_ CFU/mL).^16^^,^^17^ All the plating procedures were performed within 1 h of sampling.

### Bacterial community analysis with 16S rRNA gene sequencing

Three colonies per group (R2A plates) were analyzed. Colonies were suspended in 6 % Chelex resin (InstaGene Matrix, Bio-Rad), and DNA was extracted via centrifugation. PCR amplification of 16S rRNA genes was performed using the primers 27F (5′-GCG TAT GCA ACT TGC CTT AC-3′) and 1482R (5′-GTT TCA ACG GCA GGC TGA AC-3′) with the HotStarTaq PLUS Master Kit (QIAGEN, Hilden, Germany). Sequencing was conducted using the Sanger method (3730xl DNA Analyzer, Applied Biosystems, Waltham, MA, USA) with BigDye™ Terminator v3.1. Sequences were identified based on the GenBank database using MegaBLAST. Matches with <96 % identity or labeled as “uncultured” were excluded.

### Statistical analysis

A two-way analysis of variance (ANOVA) followed by the Tukey's honestly significant difference (HSD) multiple comparison test was used to compare FRCC and HPCs. Statistical analysis was performed using JMP Pro v17.1.0 (SAS Institute, NC, USA), with significance set at *P* < 0.05.

## Results

### Bacteria detection

No colonies were detected on the blood agar plates under either aerobic or anaerobic conditions (Fig. 1A). In contrast, the R2A agar produced abundant yellow, white, and pink colonies (Fig. 1B). Sequence analysis of 16S rRNA identified *Sphingomonas*, *Novosphingobium*, *Blastomonas*, *Rhizorhabdus*, *Methylobacterium*, and *Alphaproteobacterium* species in all the three colony types.

### DUWL contamination

The mean FRCC at the main valve was 0.39 mg/L, which decreased to 0.13 and 0.05 mg/L before the branch in the unit and at the air turbine outlet, respectively (Fig. 2C). The mean HPCs were 1.18 log_10_ CFU/mL at the main valve, 2.99 log_10_ CFU/mL before the branch in the unit, and 5.18 log_10_ CFU/mL at the air turbine outlet (Fig. 2D). As the distance from the main valve increased, the FRCC decreased, whereas the HPCs increased.

Additionally, the mean FRCC at the doctor's table was 0.09 mg/L, and the mean HPC was 3.32 log_10_ CFU/mL. As the distance from the doctor's table tap was increased, the FRCC and HPCs failed to meet the standard or target values (Fig. 2C and D).

### Examination of flushing time

After 1 min of flushing, the FRCC increased from 0.07 mg/L to 0.18 mg/L, thereby meeting the standard (Fig. 3A). The HPC before flushing was 4.77 log_10_ CFU/mL, which decreased to 3.51 log_10_ CFU/mL after 1 min of flushing. However, the HPC remained at 3.51 log_10_ CFU/mL after 4 min of flushing, thus failing to meet the target value (Fig. 3B).

### Assessment of water quality in the conventional unit and the chemically disinfected unit

Two-way ANOVA revealed the absence of interaction between the flushing time and unit type for FRCC (*P* = 0.95). In the conventional unit, the FRCC significantly increased from 0.06 ± 0.02 mg/L before flushing to 0.20 ± 0.04 mg/L after 2 min (*N* = 4, Tukey HSD test, *P* < 0.05). In contrast, the chemically disinfected unit showed no significant change after flushing (*N* = 5, Tukey's HSD test, *P* > 0.05). However, all the samples met the standard after 2 min (*N* = 5) (Fig. 4A).

For the HPCs, no significant interaction was detected between time and the unit type (*P* = 0.87). In the conventional unit, the HPCs decreased from 4.28 to 3.31 log_10_ CFU/mL after 1 min of flushing (*P* < 0.05) and further to 2.95 log_10_ CFU/mL after 2 min, thus meeting the target value (Fig. 4B). In the disinfected unit, the HPCs decreased from 3.41 to 2.68 log_10_ CFU/mL after 2 min, also meeting the target value.

Overall, compared to the conventional unit, the disinfected unit consistently showed a lower HPC, irrespective of flushing, and a more stable FRCC.

### Effect of heater temperature settings on in-tank and outlet water temperature stability

The water temperatures in the dental unit increased rapidly at the tank outlet upon flushing (Supplementary Fig. 1), indicating the swift delivery of heated water. In-tank temperatures exceeded 60 °C only at heater settings of 63 °C or higher, resulting in prolonged times at temperatures above 60 and 50 °C in the tank and at the outlet, respectively. Among all the settings, 65 °C provided the most sustained elevated temperatures at both locations. Both the maximum temperature and time spent above 60 °C in the heater tank were significantly greater at temperatures ≥65 °C than at ≤63 °C (Fig. 5B and C). Furthermore, the duration for which the tank outlet temperature remained above 50 °C was also extended at temperatures ≥65 °C compared with that at temperatures ≤62 °C (Fig. 5D).

### Impact of moderate heating on handpiece discharge water quality

**Figure 6:**
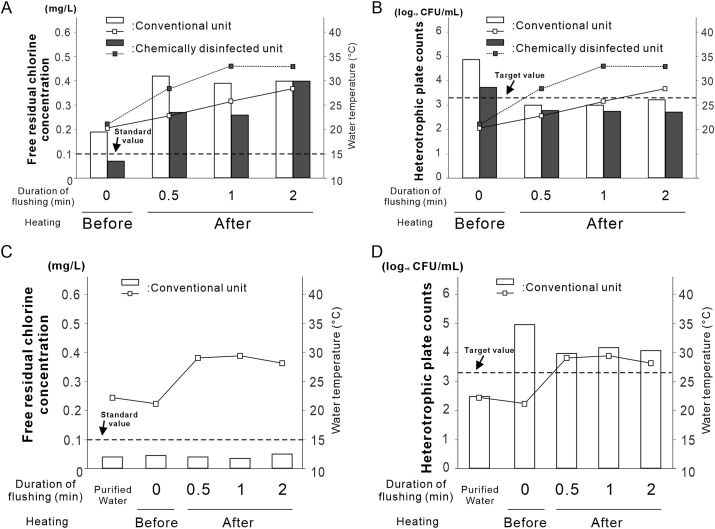
**Impact of moderate heating on handpiece discharge water quality via changes in free residual chlorine concentrations.** (A) Recovery of free residual chlorine concentration (FRCC) and increase in water temperature for each unit before and after heating. (B) Reduction in heterotrophic plate counts (HPCs) and increase in water temperature for each unit before and after heating. (C) FRCC and water temperature of purified water in the tank and air turbine discharge water before and after heating. (D) HPCs and water temperature of purified water in the tank and air turbine discharge water before and after heating. FRCC and HPCs data are shown as histograms (*N* = 1) and temperature at handpiece water discharge points at each time points before and during flushing were shown as line graphs (*N* = 2).

Before heating, the mean discharge water temperature was ∼20.7 °C in both units. After tank heating, it increased to 29.4 °C and stabilized at 30.7 °C after 2 min of flushing (Fig. 6A). In the chemically disinfected unit, the FRCC increased from 0.07 mg/L to 0.27 mg/L within 30 s of flushing, meeting the water quality standard. After 30 s, the HPCs were 3.00 and 2.77 log_10_ CFU/mL in the conventional and disinfected units, respectively—indicating that both were below the target value (Fig. 6B).

In contrast, when the water supply tank was filled with distilled water lacking free residual chlorine, the discharge temperature increased from 21.2 °C to 29.4 °C, then stabilized at 28.2 °C after 2 min of flushing (Fig. 6C). Under these conditions, the FRCC remained at 0.05 mg/L and did not meet the standard. The HPCs slightly decreased from 4.95 to 3.96 log_10_ CFU/mL after 30 s of flushing but increased to 4.06 log_10_ CFU/mL after 2 min, remaining above the target value (Fig. 6D).

## Discussion

Heterotrophic bacteria that grow under low-nutrient and low-temperature conditions are important indicators of water quality. Although generally non-pathogenic, their presence at high concentrations may pose risks to immunocompromised individuals.^18^, ^19^, ^20^ In this study, no general bacteria were detected (Fig. 1A); however, heterotrophic bacteria, such as *Sphingomonas* and *Methylobacterium*, similar to those found in portable dental units,^17^ were identified (Fig. 1B). The Japanese Ministry of Health sets a non-regulatory target of ≤2,000 CFU/mL for HPCs in water systems. To characterize the bacterial populations in this study, three representative colonies (yellow, white, and pink) were selected from R2A agar plates for 16S rRNA gene sequencing to reflect visually distinct and recurrent morphotypes. These colonies were consistently observed across samples and thus considered representative of dominant heterotrophic bacterial populations in DUWLs. However, the limited number of colonies analyzed may have excluded non-dominant species, which could contribute to the overall microbial diversity. To fully elucidate the bacterial community composition in DUWLs, a comprehensive, culture-independent approach, such as metagenomic analysis, would be required. This represents an important avenue for future investigation.

DUWLs have long, narrow, and complex waterlines that encourage microbial growth owing water stagnation and chlorine depletion. We found that the FRCC decreased, whereas the HPCs increased as water traveled away from the main valve (Fig. 2C and D), especially in the section between the doctor's table and handpiece. This study highlights the need for location-specific control strategies by underscoring the contribution of the internal piping structure of dental units to the risk of bacterial contamination. In addition, although the HPCs did not reach the target value even after 4 min of flushing, 1 min of flushing (∼55 mL) was sufficient to restore the FRCC, even in the handpiece discharge water to meet the tap water quality standard (Fig. 3A and B). This suggests that effective water quality control can be achieved with a shorter flushing duration than the 3–5 min recommended by the Japanese Association for Dental Science, provided that supplemental measures are employed to further reduce HPCs. Therefore, the application of moderate heating is particularly significant because it enhances chlorine efficacy and further reduces bacterial counts without the introduction of chemical agents.

The chemically disinfected unit showed consistently lower HPC values compared to those of the conventional unit, although the increase in FRCC was modest (Fig. 4A and B). External disinfection devices may compensate for conventional units that lack internal disinfection systems.^21^ Nonetheless, flushing remains essential, as disinfectants alone may not maintain acceptable FRCC or HPC values. Drawbacks such as pipe corrosion, blockages,^22^^,^^23^ altered water taste,^24^ and chemical reactions (e.g., H_2_O_2_ reducing chlorine levels)^25^ must also be considered.

Moderate heating has been assessed as a chemical-free method for microbial control. At 65 °C, in-tank temperatures remained above 60 °C, and outlet temperatures above 50 °C during flushing (Fig. 5B–D and Supplementary Fig. 1), meeting international guidelines for Legionella control.^26^^,^^27^ Although effective, high temperatures may damage the DUWL components and pose scalding risks.^28^ Thus, 65 °C was selected as the optimal temperature. Following heating and flushing for 30 s, the HPCs content decreased below the target value in both the units (Fig. 6A and B). However, when distilled water was used in the conventional unit, the FRCC did not increase. The HPCs remained above the target (Fig. 6C and D), suggesting that heating alone was insufficient without residual chlorine. Therefore, applying combination of heating and residual disinfectants is a practical approach.

Although several limitations are observed in the evaluation of biofilm responses, structural variations across units, and durability of DUWL components under repeated heating, this study demonstrated that moderate heating combined with flushing can improve water quality without chemicals, making it safe and cost-effective. It satisfies both FRCC and HPC targets and can help reduce the reliance on chemical disinfectants, thereby minimizing the development resistance. Additionally, a limitation of this study is the small sample size (*N* = 1 or 2) in certain experiments (Figs. 2C and D, Figure 3, Figure 6). These experiments involved the disassembly of clinically operating dental units, and the installation of internal heating devices required substantial modification of the internal structures. Owing to practical constraints, including limited access to the units, clinical workflow considerations, and ethical concerns, conducting experiments with larger sample sizes was not feasible. While the results exhibit consistent trends, the statistical robustness and generalizability of the findings may require further verification in future studies.

Although this study did not directly assess biofilm detachment, the observed reduction in HPCs may partially reflect the disruption of biofilm-associated bacteria. For instance, the extracellular polymeric substances (EPS) of *Klebsiella pneumoniae*, a representative heterotrophic bacterium, have been reported to exhibit reduced mechanical stability at temperatures ≥45 °C, with diminished surface attachment at 50 °C.^29^ Moreover, the yield stress of biofilms of *Staphylococcus epidermidis*, a common resident skin bacterium, decreases by ∼90 % at 60 °C, accompanied by a >70 % reduction in bacterial viability, indicating increased susceptibility to thermal detachment.^30^ Once detached, planktonic bacteria may become more vulnerable to residual chlorine, as supported by the elevated FRCC following heating. To clarify these interactions, future studies should directly evaluate biofilm removal and composition by analyzing the EPS structure and mechanical behavior across multiple bacterial species under various thermal conditions. Such investigations will strengthen the scientific foundation for implementing thermal disinfection strategies in DUWLs and other water-based infrastructures.

In conclusion, flushing is essential for improving the DUWL water quality and restoring FRCC to meet legal standards. However, maintaining HPCs below the target levels remains challenging. Chemical disinfection helps suppress bacterial growth, but does not restore FRCC to meet legal standards. Moderate heating further enhances disinfection efficacy, making it a practical, non-chemical strategy to achieve both FRCC compliance and bacterial control.

## Declaration of competing interest

Masahiro Yamada, Jumpei Washio, Nobuhiro Takahashi and Hiroshi Egusa are co-inventors of a granted patent related to the content of this manuscript. This study was partially supported by GC Corporation, and GC Corporation's products were used in the study. The other authors declare no conflict of interest.
