# Supplementary material for: Moderate heating of waterline improves dental unit water quality by activating bactericidal properties of tap water
Source: J Dent Sci. 2025 Jun 6;21(1):216–24. doi: 10.1016/j.jds.2025.05.023 (PMC12825499; doi:10.1016/j.jds.2025.05.023)
Supplement: Multimedia component 1 [file mmc1.docx]

**Article category**: Original Article

**Article title**: Moderate heating of waterline improves dental unit water quality by activating bactericidal properties of tap water

**Author names**: Kunihiro Fushimi ^a^, Masahiro Yamada ^a, b^*, Jun Watanabe ^a^, Jumpei

Washio ^c^, Nobuhiro Takahashi ^c, d^, Hiroshi Egusa ^a, e**^

**Affiliations**

^a^ *Division of Molecular and Regenerative Prosthodontics, Tohoku University Graduate School of Dentistry, Sendai, Japan*

^b^ *Division of Mechanobiology and Biomedical-Dental Engineering, Tohoku University Graduate School of Biomedical Engineering, Sendai, Japan*

^c^ *Division of Oral Ecology and Biochemistry, Tohoku University Graduate School of Dentistry, Sendai, Japan*

^d^ *Global Strategy Office, Tohoku University, Sendai, Japan*

^e^ *Center of Excellence for Dental Stem Cell Biology, Faculty of Dentistry, Chulalongkorn University, Bangkok, Thailand*

*****Corresponding author. Division of Molecular and Regenerative Prosthodontics, Tohoku University Graduate School of Dentistry, 4-1 Seiryo-machi, Aoba-ku, Sendai, 980-8575, Japan.

******Corresponding author. Division of Molecular and Regenerative Prosthodontics, Tohoku University Graduate School of Dentistry, 4-1 Seiryo-machi, Aoba-ku, Sendai, 980-8575, Japan.

E-mail addresses: yamamasa@tohoku.ac.jp (M. Yamada), egu@tohoku.ac.jp (H. Egusa)

**Running title**: Heat-assisted flushing for dental unit waterlines

**
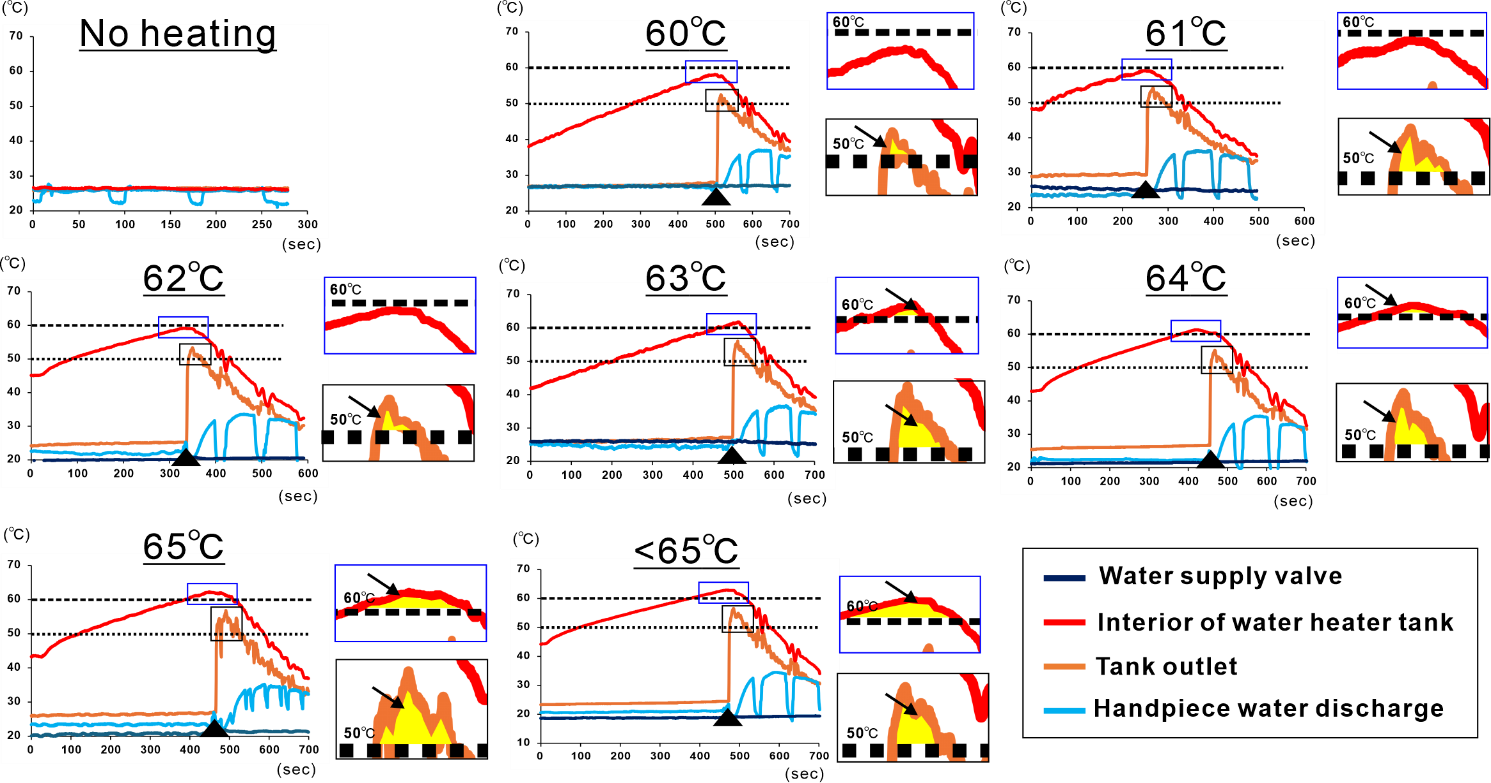
**

**Supplementary Figure 1 Impact of moderate heating on water temperature in a dental unit**

A water-heating tank (GC Corporation, Tokyo, Japan) was integrated into the conventional dental unit. The heater temperature was adjusted in 1℃ increments between 60 and 69 ℃. Following heating, flushing was performed for 30 s, 1 min, or 2 min. To determine the optimal heating temperature, the water temperature was monitored at four key locations along the dental unit waterline: (1) water supply valve, (2) interior of the water heater tank, (3) tank outlet, and (4) handpiece discharge point. Measurements were taken under various heater settings, including no heating and settings ≥65 °C.

The line graphs display the time-course changes in the water temperature at each monitoring point under each heater setting. The black triangles mark the start of flushing. Dashed lines indicate 60  (heater tank) and 50 °C (tank outlet) based on the international guidelines for Legionella control. The enlarged panels show the temperature transitions near these thresholds. The black arrows denote the duration for which the temperatures exceeded the respective threshold values.

After flushing commenced, the heater tank temperature gradually decreased as the heated water was discharged. The in-tank temperature exceeded 60 °C only when the heater temperature was set to 63 °C or higher. In contrast, the lower settings did not reach this threshold. Heater settings of 63 °C or higher extended the duration for which the tank outlet temperature remained above 50 °C. Among all the tested conditions, the 65 °C setting maintained temperatures above both critical thresholds for the longest duration.
